# Bioinformatics Analysis Reveals a Novel Prognostic Model for Esophageal Squamous Cell Carcinoma

**DOI:** 10.7150/ijms.93423

**Published:** 2024-05-05

**Authors:** Huan Yang, Yang Chen, Xiancong Huang, Yixuan Gu, Zhongjian Chen, Weimin Mao

**Affiliations:** 1 The Second Clinical Medical College, Wenzhou Medical University, Wenzhou, 325088, China; 2 Department of Medical Oncology, the Second Clinical Medical College of Zhejiang Chinese Medical University, Hangzhou, Zhejiang, 310053, China.; 3 Zhejiang Key Laboratory of Diagnosis and Treatment Technology on Thoracic Oncology, Hangzhou, Zhejiang, 310022, China.; 4 The Cancer Research Institute, Zhejiang Cancer Hospital, Hangzhou, Zhejiang, 310022, China.

**Keywords:** Esophageal squamous cell carcinoma (ESCC), bioinformatics analysis, prognosis, B3GNT3

## Abstract

**Background**: Esophageal squamous cell carcinoma (ESCC), a gastrointestinal cancer, is associated with poor prognosis. Prognostic models predict the likelihood of disease progression and are important for the management of patients with ESCC. The objective of this study was to develop a prognostic model for ESCC using bioinformatics analysis.

**Methods**: Two transcriptome microarray Gene Expression Omnibus ESCC datasets (GSE53624 and GSE53622) were analyzed using bioinformatics methods. Differentially expressed genes (DEGs) were identified using the R package limma, and genes associated with survival outcomes in both datasets were identified by Kaplan-Meier analysis. Genes with diagnostic or prognostic value were selected for further analysis, and hazard ratios and their relationship with pathological TNM (pTNM) staging were investigated using univariate and multivariate Cox analysis. After selecting the independent factors from pTNM staging, Cox analysis and nomogram plotting were performed. The ability of the model to stratify risk and predict survival was evaluated and compared with the pTNM staging system to determine its potential clinical value. Key genes were analyzed by immunohistochemistry and RT-PCR.

**Results**: Four candidate genes (B3GNT3, MACC1, NELL2, and USH1G) with prognostic value were identified from the two transcriptome microarray datasets. Age, pTNM stage, and B3GNT3, MACC1, and NELL2 were identified as independent factors associated with survival in the multivariate Cox analysis and used to establish a prognostic model. The model demonstrated significantly higher accuracy in predicting 3-year survival than the pTNM staging system and was useful for further risk stratification in patients with ESCC. B3GNT3 was significantly downregulated in ESCC tumor tissues and negatively associated with lymph node metastasis. Bioinformatics analysis indicated that B3GNT3 may play a role in immune regulation by regulating M2 macrophages.

**Conclusion**: This study developed a new prognostic model for ESCC and identified B3GNT3 as a potential biomarker negatively associated with lymph node metastasis, which warrants further validation.

## 1. Introduction

Esophageal cancer (ESCA) poses a substantial global health burden, ranking eighth in incidence and sixth in cancer-related mortality worldwide 1. There are two main types of ESCA, esophageal adenocarcinoma (EAC) and esophageal squamous cell carcinoma (ESCC), and the latter accounts for 95% of cases in China. The main treatment for ESCC is surgical resection with lymphadenectomy; however, despite advances in management and multidisciplinary care, the 5-year survival rate remains poor 2. The pTNM staging system is limited by its lack of information, subjectivity, and inability to account for tumor heterogeneity 3. Therefore, new prognostic models including multiple factors are needed to predict prognosis more accurately and to improve treatment guidance.

Transcriptomic data analysis is a potent prognostic tool in the field of cancer research that can facilitate the identification of gene expression signatures linked to diverse clinical outcomes, such as overall survival and disease-free survival. Prognostic models developed using these signatures can predict a patient's risk of disease recurrence or progression, thereby providing information for treatment decisions and cancer research 4. An increasing number of studies use bioinformatics analysis to develop prognostic models or biomarkers for ESCC using the widely used open-access database, the Gene Expression Omnibus (GEO), for transcriptomic data analysis 5. A prognostic model for ESCC based on transcriptomic data was developed by Zheng *et al.* The model comprised four genes and demonstrated good predictive accuracy for ESCC prognosis 6. Similarly, Guo *et al.* developed a prognostic model for ESCC that included seven immune-related genes. This model also showed good predictive accuracy for ESCC prognosis 7. Validation of potential prognostic biomarkers is crucial to ensure their reliability and accuracy, because false-positive results may occur in bioinformatics studies due to a lack of validation of protein expression levels.

The objective of this study was to develop a novel prognostic model for ESCC using bioinformatics analysis, which identified four candidate genes with prognostic value. The prognostic model based on age, pTNM stage, and B3GNT3, MACC1, and NELL2 showed greater accuracy in predicting 3-year survival than the pTNM staging system. A potential biomarker, B3GNT3, was identified as a key gene that was negatively associated with lymph node metastasis and positively correlated with prognosis.

## 2. Material and methods

### 2.1. GEO dataset and probe re-annotation

The transcriptomic and clinical data of patients diagnosed with ESCC were obtained from the publicly accessible GEO database (https://www.ncbi.nlm.nih.gov/geo/). Cases lacking clinical survival information were excluded from the study. Two transcriptome microarray datasets, GSE53624 and GSE53622, which were generated using the Agilent-038314 CBC Homo Sapiens lncRNA + mRNA microarray V2.0 (http://www.genomics.agilent.com/), were chosen for analysis in this research.

The dataset GSE53624 contains 119 paired cancer and normal tissue samples, whereas GSE53622 contains 60 pairs of cancer-matched normal tissue samples. The two datasets included complete clinical information such as age, sex, drinking habits, tumor grade, T stage, N stage, pTNM stage and survival status and time.

ESCC cases were restaged according to T and N stages following the guidelines of the 8th edition of the American Joint Committee on Cancer (AJCC). The key clinicopathological characteristics of the patients are summarized in **Table S1**. The GSE53624 and GSE53622 datasets were analyzed separately, and genes showing the same trend were considered candidate genes.

Probe re-annotation was performed according to the method reported by Guo *et al.* 8. In brief, the GPL18109 probe set sequences for the Agilent-038314 CBC Homo Sapiens lncRNA + mRNA microarray V2.0 were obtained from the Agilent website. The mRNA expression profile data were obtained by re-annotating microarray probes using SeqMap software based on the sequences of the probe sets and the protein-coding transcript sequences in GENCODE (GRCh38, release 40). Probes that matched more than one gene were removed, resulting in 32208 mRNA probes. Annotation was performed for both GSE53624 and GSE53622, and a mean value was used when multiple probes matched one gene. Overall, 17746 genes were annotated in both datasets.

### 2.2. Identification of differentially expressed genes

To identify differentially expressed genes (DEGs) in ESCC, R package limma (version 3.54.2) was used to analyze the differences in gene expression between cancer and normal tissues for the GSE53624 and GSE53622 datasets, separately. The criteria for DEGs in this study were an adjusted *p* < 0.05 and |log_2_(fold change)| > 1. The difference in gene expression between ESCC and normal tissues was analyzed by generating a heat map using the R package pheatmap (version 1.0.12). Venn plots showing the same changing trend (up- or downregulated) in the GSE53624 and GSE53622 datasets were used to identify candidate DEGs in ESCC.

### 2.3. Functional enrichment analysis

The Gene Ontology database (GO, http://geneontology.org/), encompassing molecular function (MF), biological process (BP), and cellular component (CC) data, was used to analyze the biological mechanisms associated with DEGs. The Kyoto Encyclopedia of Genes and Genomes (KEGG; https://www.kegg.jp/) database was used to determine the functional roles and biological correlations of the DEGs identified. Visualization of GO and KEGG pathway data was accomplished using the Cluster Profiler R package (version 4.6.2). Statistical significance was determined using the cut-off criteria of* p* < 0.05 and false discovery rate (FDR) < 0.05.

### 2.4. Survival analysis

To investigate the relationship between gene expression levels and overall survival in ESCC patients, the population was divided into two groups according to median gene expression levels: high expression and low expression groups. Kaplan-Meier survival curves were constructed for each group, and the differences in survival between the two groups were analyzed by the log-rank test. Univariate Cox analysis was performed, and the hazard ratio (HR) was calculated for each gene. Survival analysis was performed separately in both the GSE53624 and GSE52622 datasets, and candidate prognostic genes were selected based on log-rank *p* < 0.05 and the same HR trend (HR > 1 or < 1) between the two datasets. A Venn diagram was utilized to visualize the DEGs with prognostic value shared between the GSE53624 and GSE52622 datasets. The analyses were performed using survival package (version 3.5-5) and survminer package (version 0.4.9) in R.

### 2.5. Development of prognostic model

Univariate Cox analysis was used to investigate candidate genes and clinical variables, whereas multivariate Cox analysis was performed to confirm the independent predictive potential of the candidate genes. A risk score was calculated for each patient by incorporating the identified independent prognostic factors and key clinical characteristics (age, pTNM stage) using multivariate Cox analysis. Patients were categorized into two groups according to the median risk score, and the prognostic differences between the groups were assessed using Kaplan-Meier curves. The 3-year overall survival was predicted by ROC analysis of the risk scores and pTNM staging using the R software package pROC (version 1.18.0). The area under the curve (AUC) of the ROC between the model and stage was compared using the DeLong test 9.

### 2.6. Quantitative real-time polymerase chain reaction

Total RNA was isolated from 10 pairs of ESCC and normal tissues using an RNA isolation kit (Macherey-Nagel, Germany), followed by cDNA synthesis using the PrimeScript RT reagent Kit with gDNA Eraser (Takara Biomedical Technology Co., Ltd., Beijing, China). Real-time quantitative polymerase chain reaction (RT-PCR) was performed on an Applied Biosystems 7500 real-time PCR system utilizing the SYBR Green method. Primer sequences for RT-PCR are listed in **Table 1**. The data were analyzed using paired t-tests, and error bars were labeled with Standard Error of the Mean (SEM). Graphs were plotted using GraphPad Prism (version 6.01). Detailed patient information on tissues is provided in **Table S2**.

### 2.7. Immunohistochemistry staining

Paraffin-embedded ESCC tissues (n=154) were obtained from patients with pathologically proven ESCC who received esophagectomy in Zhejiang Cancer Hospital. In addition, 33 paraffin-embedded normal esophageal mucosa samples were used as controls. The experiments, along with all pertinent details, received approval from the Ethics Committee of Zhejiang Cancer Hospital. The study was conducted in adherence to pertinent guidelines and regulations. Written informed consent was obtained from the patients, allowing their anonymized clinical information to be included in this article. Immunohistochemistry (IHC) staining analysis was performed according to the antibody instructions. The primary antibodies used were MACC1 (Proteintech, China, 1:100), NELL2 (Proteintech, China, 1:100), and B3GNT3 (Proteintech, China, 1:100). Staining was independently assessed by qualified pathologists based on the percentage of positive cells and the staining intensity. The final immunoreactivity score (IRS) was obtained by multiplying the staining intensity score by the percentage of positive cells. IHC data were analyzed using unpaired t-tests, and error bars were labeled with standard deviation (SD). Plots were generated using GraphPad Prism (version 6.01). The detailed patient clinical information from tissue microarrays is presented in **Table S3.** All tissues were restaged according to the 8th edition of the AJCC guidelines, following pTNM staging.

### 2.8. Immune Environment Evaluation and Gene Set Enrichment Analysis

The extent of infiltration for 28 immune cell types was calculated using the single-sample gene set enrichment analysis (ssGSEA) method from the R package GSVA (version 0.4.9). This calculation was based on the expression levels of genes in 28 published gene sets specifically curated for immune cells 10, 11. The correlation between B3GNT3 expression and immune cells was determined using Spearman's rank correlation. In addition, Gene Set Enrichment Analysis (GSEA) was used to analyze the biological processes associated with B3GNT3 in ESCC. The genes were organized into two clusters according to the expression levels of B3GNT3. These clusters were then sorted using differential analysis, followed by KEGG enrichment analysis using the R ClusterProfiler package (version 4.6.2). Significant pathway enrichment was defined as p < 0.05 and q < 0.25.

## 3. Results

**Figure 1:**
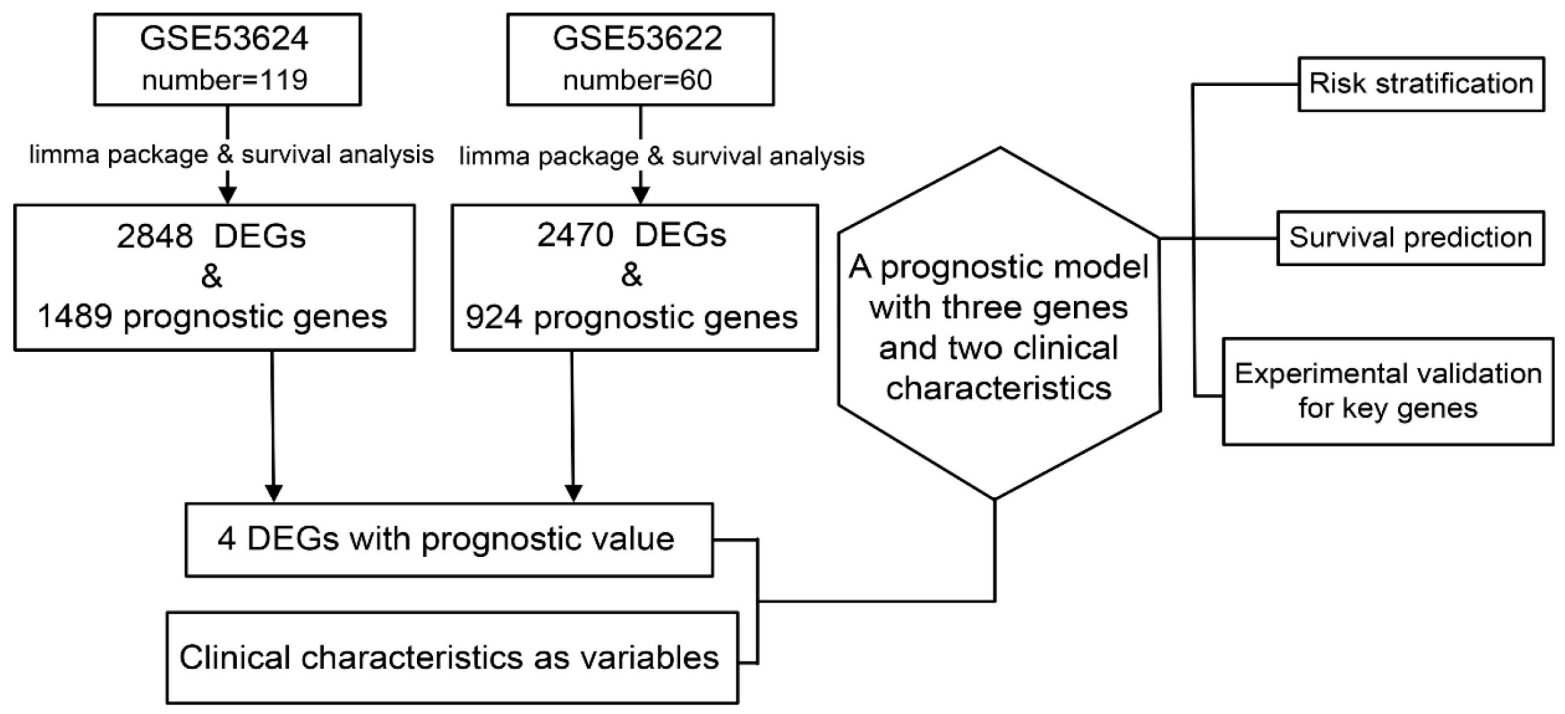
Data preparation, processing, and bioinformatics analysis flowchart.

The study design is described in **Figure 1**.

### 3.1. DEGs in ESCC

**Figure 2:**
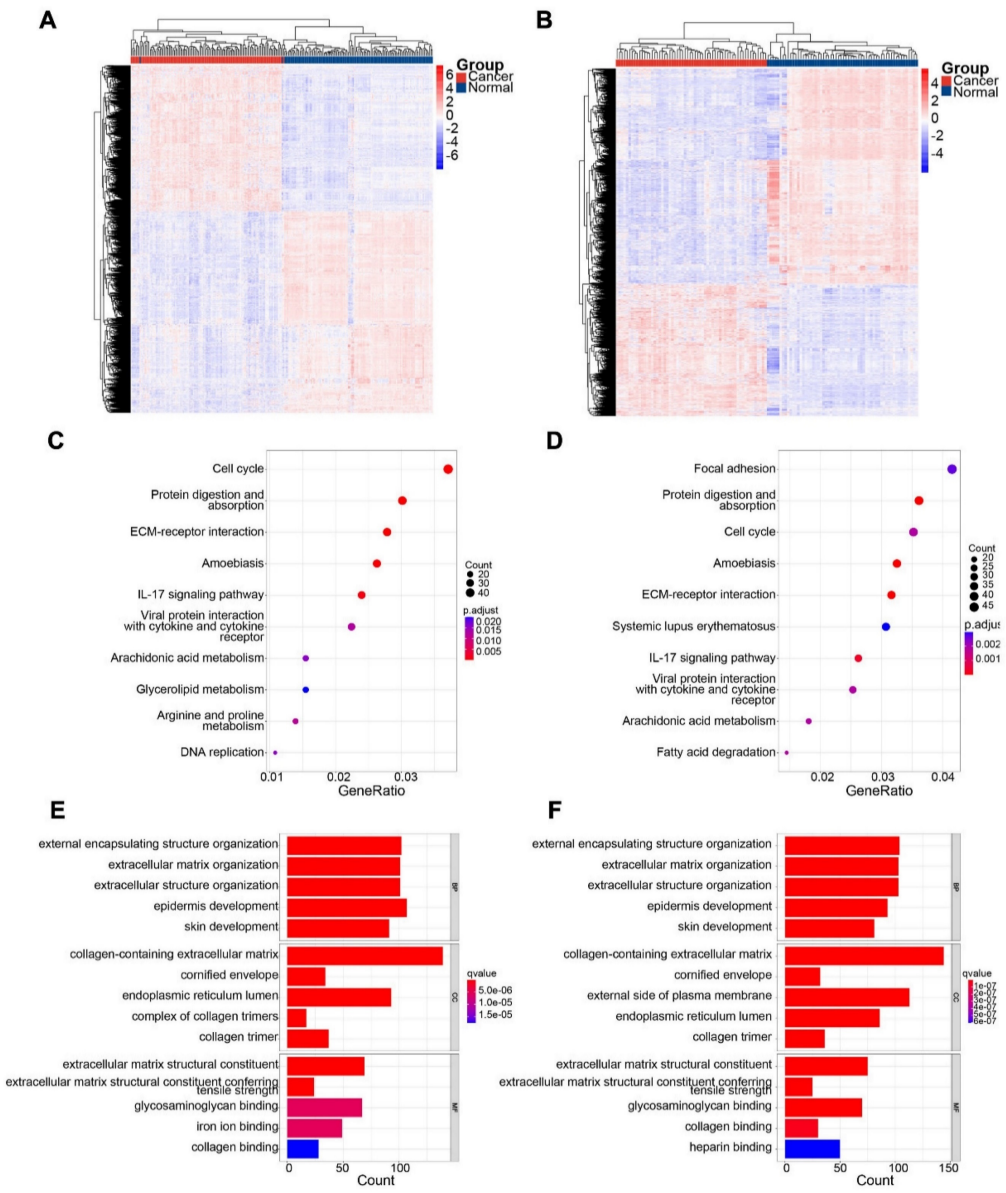
Identification of DEGs in the two subsets and significant functional enrichment. (A) and (B) show heatmaps of GSE63524 and GSE53622, respectively. (C) and (D) show KEGG analyses of GSE63524 and GSE53622, respectively. (E) and (F) show GO analyses of GSE63524 and GSE53622, respectively.

**Figure 3:**
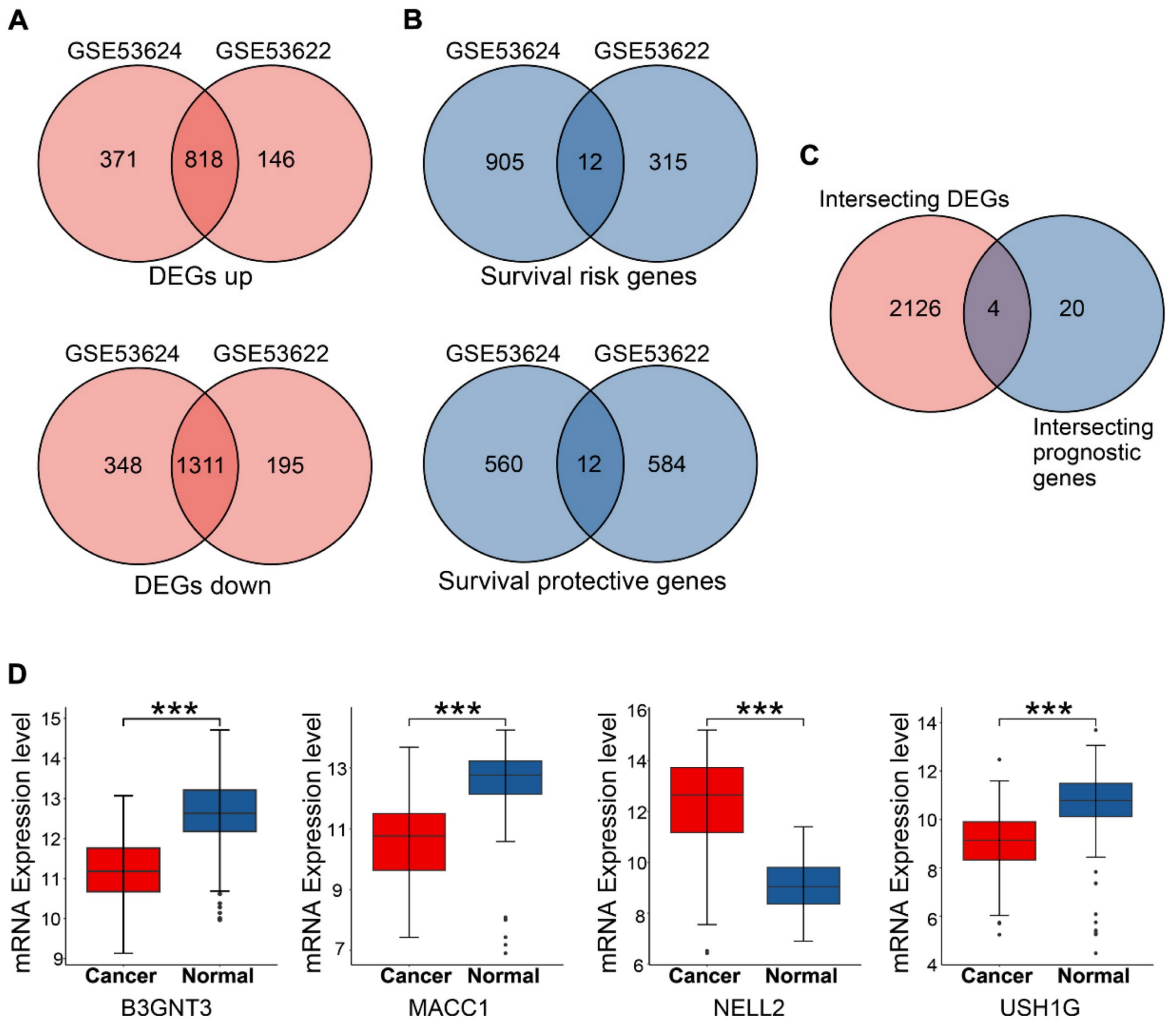
Overlapping genes in GSE53624 and GSE53622 were identified, and the expression of four candidate genes was assessed. (A) Up- and down-regulated DEGs in the two subsets. (B) Risk and protective genes associated with prognosis in the two subsets. (C) Venn plot showing four DEGs with prognostic value. (D) Boxplots showing the mRNA expression levels of four candidate genes in 179 patients. The p values are indicated using asterisks (* p < 0.05, ** p < 0.01, *** p < 0.001, **** p< 0.0001).

In the GSE53624 subset, 1189 genes were upregulated and 1659 genes were downregulated. In the GSE53622 subset, 964 genes were upregulated and 1506 genes were downregulated. The expression patterns of DEGs in GSE53624 and GSE53622 were visualized using a heat map (**Figure 2A** and **B**). The underlying biological mechanisms of DEGs in the two datasets were analyzed using KEGG and GO functional enrichment analyses. KEGG functional analysis revealed that the two subsets of DEGs were enriched in seven identical pathways of cell cycle, protein digestion and absorption, ECM-receptor interaction, amoebiasis, IL-17 signaling, viral protein interaction with cytokine and cytokine receptor, and arachidonic acid metabolism (**Figure 2C** and** D**). Similarly, DEGs from both datasets were enriched for the same GO BPs, and the analyses revealed the functional categories in the BPs associated with external encapsulating structure organization, extracellular matrix organization, extracellular structure organization, and skin development. Regarding CC, there was significant enrichment in processes related to collagen-containing extracellular structure, collagen-containing extracellular matrix, cornified envelope, endoplasmic reticulum lumen, and collagen trimer. In terms of MF, the DEGs were enriched in processes associated with extracellular matrix structural constituent, extracellular matrix structural constituent conferring tensile strength, glycosaminoglycan binding, and collagen binding (**Figure 2E** and** F**). The Venn diagram indicated that 817 genes were consistently upregulated, whereas 1311 genes were consistently downregulated in both datasets (**Figure 3A**).

### 3.2. Prognostic genes in ESCC

Univariate Cox analysis and log-rank tests were performed for both subsets to identify genes with potential prognostic significance. Genes with p < 0.05 and HR > 1 were classified as risk genes, whereas genes with p < 0.05 and HR < 1 were classified as protective genes. The GSE53624 subset included 917 risk genes and 572 protective genes, whereas the GSE53622 subset had 327 risk genes and 596 protective genes. In both subsets, 12 risk genes and 12 protective genes were identified (**Figure 3B**). HRs and p values for the genes screened for prognostic relevance are provided in **Table 2**. To further identify candidate genes, DEGs and prognostic genes were screened for additional potential genes. B3GNT3, MACC1, NELL2, and USH1G were identified as DEGs with potential prognostic value (**Figure 3C**). The box plots show the expression of these four candidate genes in 179 patients (**Figure 3D**).

### 3.3. Construction of prognostic models

**Figure 4:**
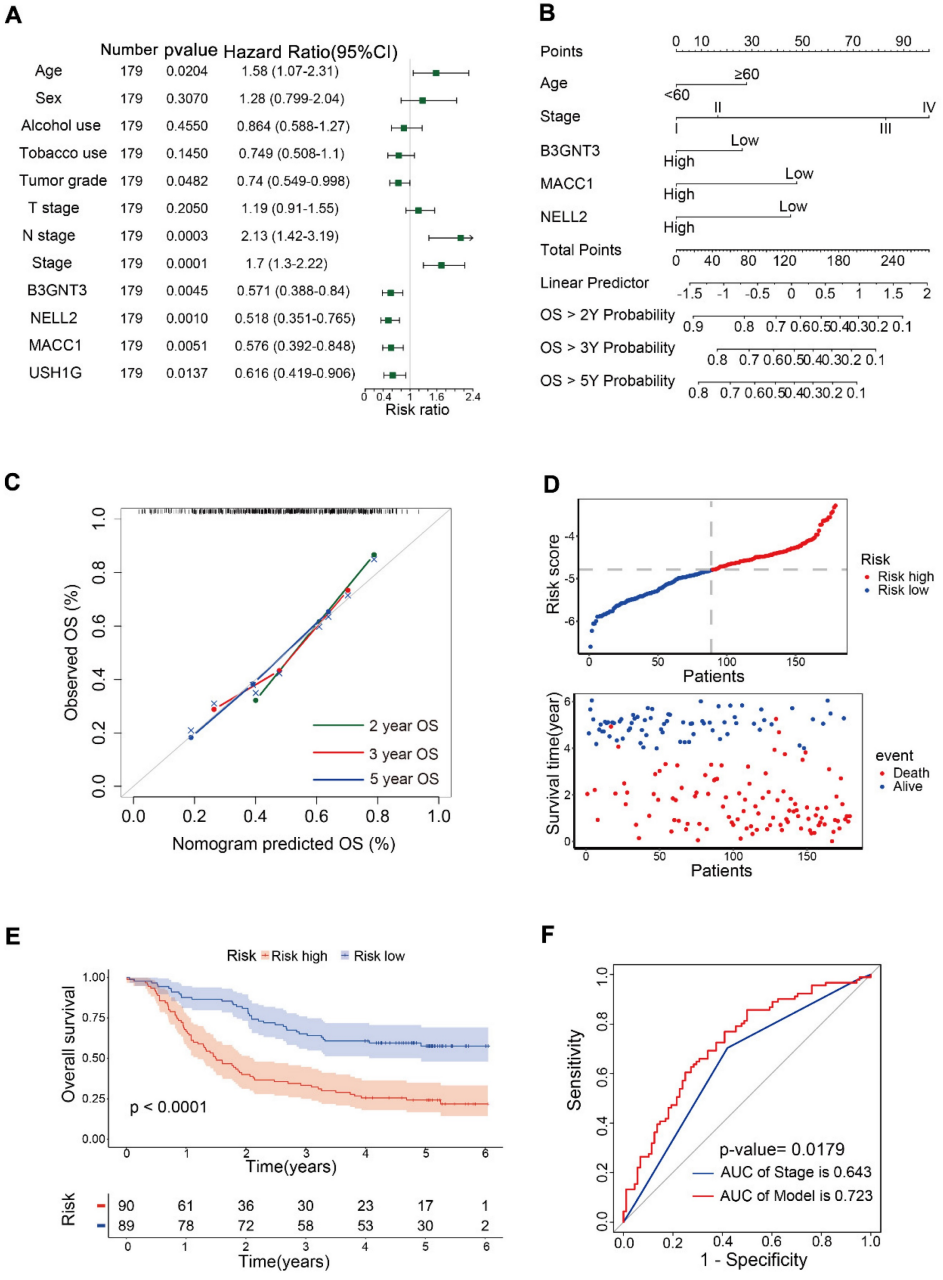
Prognostic models were constructed in 179 patients. (A) Forest plot showing univariate Cox analysis of four candidate genes and eight clinical characteristics. (B) Nomogram for predicting 2-, 3-, and 5-year overall survival in 179 patients with ESCC. Nomogram calibration curves (C) for predicting 2-, 3-, and 5-year OS. The risk score distribution and scatter plot (D) show the distribution of risk scores and corresponding survival status for each patient. Kaplan-Meier curve (E) for ESCC patients stratified by the risk score with a median-split. ROC curves (F) were used to evaluate the predictive accuracy of 3-year survival rates in the prognostic model and stage, followed by the Delong test to compare the performance of the two models.

Univariate Cox analysis was performed for 12 variables, including B3GNT3, MACC1, NELL2, USH1G, age, sex, alcohol use, tobacco use, tumor grade, T stage, N stage and pTNM stage. Age, N stage, and pTNM stage were identified as risk factors, whereas tumor grade, and B3GNT3, MACC1, NELL2, and USH1G were identified as protective variables (**Figure 4A**). Multivariate Cox analysis revealed that B3GNT3, MACC1, and NELL2 were independent prognostic factors. Therefore, B3GNT3, MACC1, NELL2, age, and pTNM staging were used to further construct prognostic models using multivariate Cox analysis. Each patient's risk score was derived using the following formula: risk score = age × 0.41354 + stage × 0.53042 + expression of B3GNT3 × (-0.25258) + expression of NELL2 × (-0.17000) + expression of MACC1 × (-0.14138). A nomogram was generated to predict patient survival at 2, 3, and 5 years (**Figure 4B**). Meanwhile, the calibration curves for 2-, 3- and 5-year overall survival probabilities indicated the accuracy of this nomogram (**Figure 4C**). Furthermore, as the risk score increased, there was a corresponding increase in the number of deaths (**Figure 4D**). Kaplan-Meier curves generated using the median risk score values revealed that patients in the low-risk group had higher survival rates than those in the high-risk group (**Figure 4E**). ROC curve analysis was performed to calculate the area under the curve (AUC) for both the prognostic model (AUC = 0.723) and the pTNM staging system (AUC = 0.643). A comparison of AUCs using the method proposed by DeLong *et al.* indicated that the model exhibited superior predictability to the pTNM staging system (**Figure 4F**).

### 3.4. Risk stratification for ESCC

**Figure 5:**
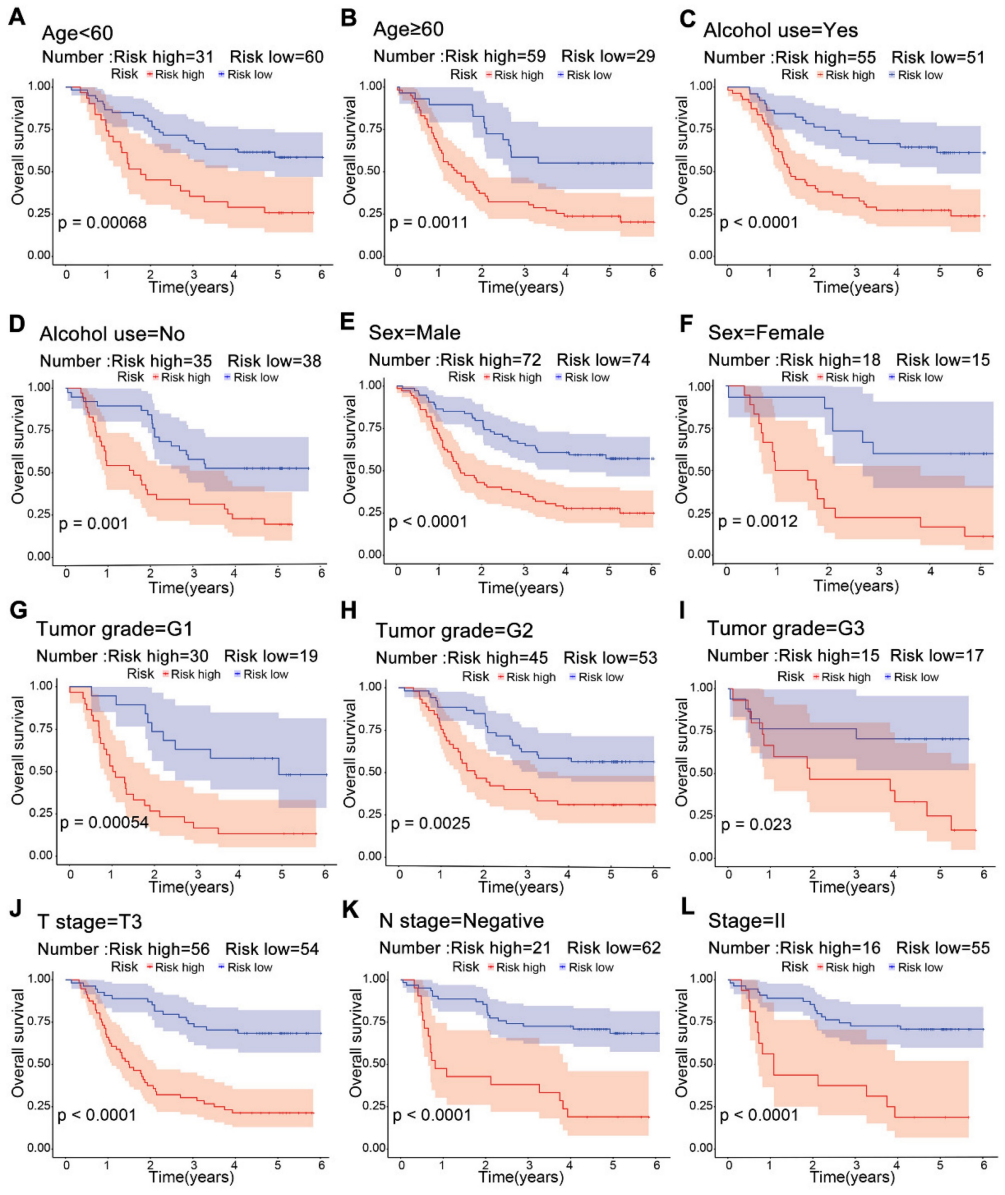
Kaplan-Meier survival curves for risk groups in clinical subgroups. Risk stratification based on age (A, B), alcohol use (C, D), sex (E, F), tumor grade G1-G3 (G-I), T3 stage (J), lymphatic node metastasis negativity (K), and stage II (L).

Significant differences in survival were observed between the high-risk and low-risk groups across multiple clinical subgroups. Kaplan-Meier survival analysis of subgroups according to age, alcohol use, sex and tumor grade showed a worse prognosis in the high-risk group (**Figure 5A-F**). For T3, negative lymph node and stage II, patients in the low-risk group had a higher survival rate (**Figure 5J-L**). This suggests that risk scores from the model can predict prognosis, which may help in clinical decision-making.

### 3.5. Experimental verification of three genes in ESCC tissue

**Figure 6:**
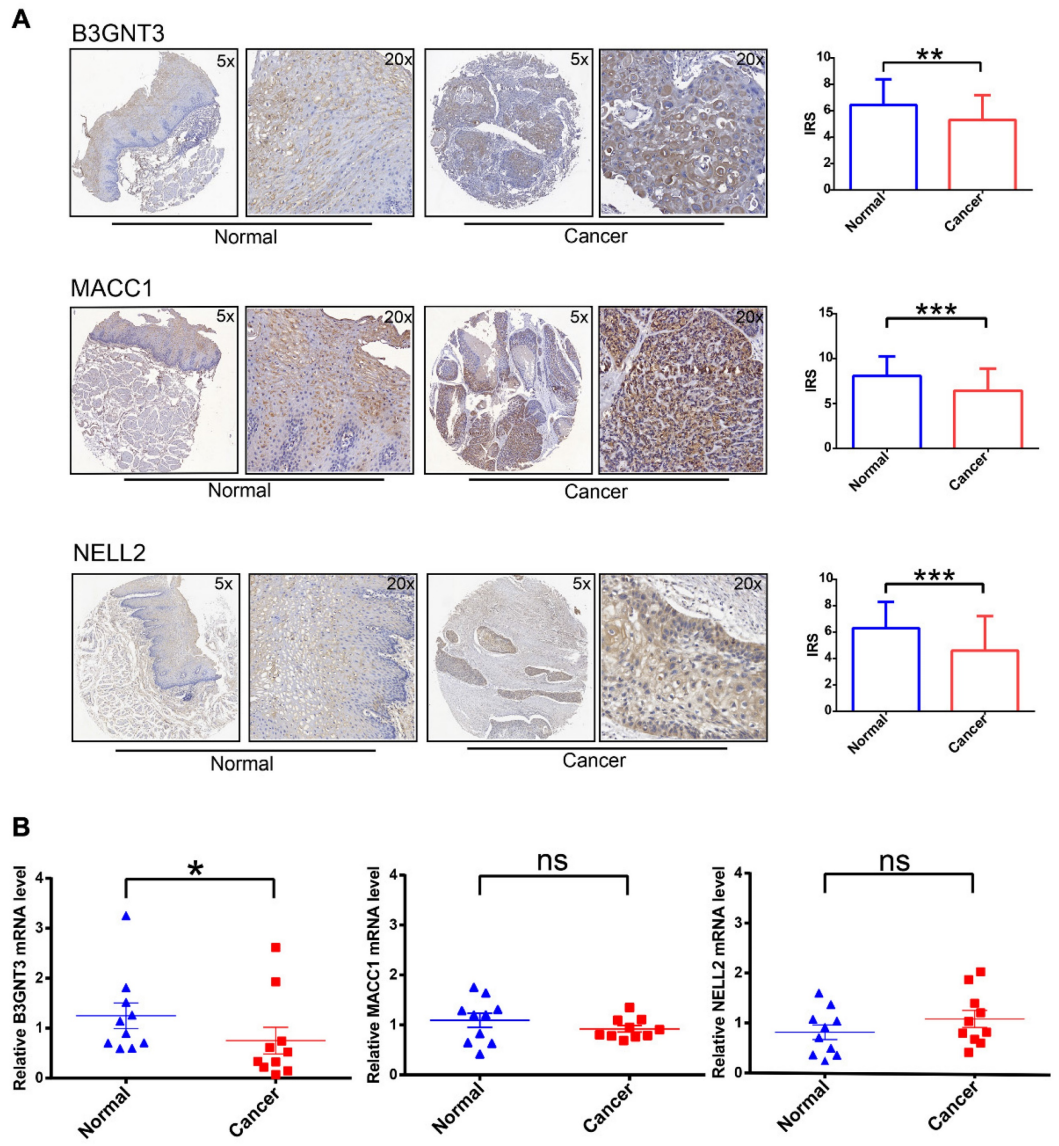
IHC and RT-PCR validation of three key genes in cancerous tissues and normal tissues. (A) Representative images of B3GNT3, MACC1, and NELL2 expression levels in cancerous tissues and normal tissues detected by immunohistochemical staining. (B) show the relative mRNA expression levels of B3GNT3, MACC1, and NELL2 in cancerous tissues and normal tissues. The p values are indicated using asterisks (ns, no significantly, * p < 0.05, ** p < 0.01, *** p < 0.001, **** p< 0.0001).

To increase the credibility of the bioinformatic analysis, we analyzed the protein expression level of the three signatures (B3GNT3, MACC1, NELL2) in cancerous tissues compared with normal tissues by IHC. B3GNT3, MACC1, and NELL2 showed significantly higher expression in normal tissues than in tumor tissues (**Figure 6A**). In addition, RT-PCR was performed to quantify the mRNA expression levels of these genes in paired cancerous and normal tissue samples (**Figure 6B**). These analyses at the transcriptome and protein levels confirmed that B3GNT3 is a key representative gene.

### 3.6. Association of B3GNT3 expression in ESCC tissues with survival

**Figure 7:**
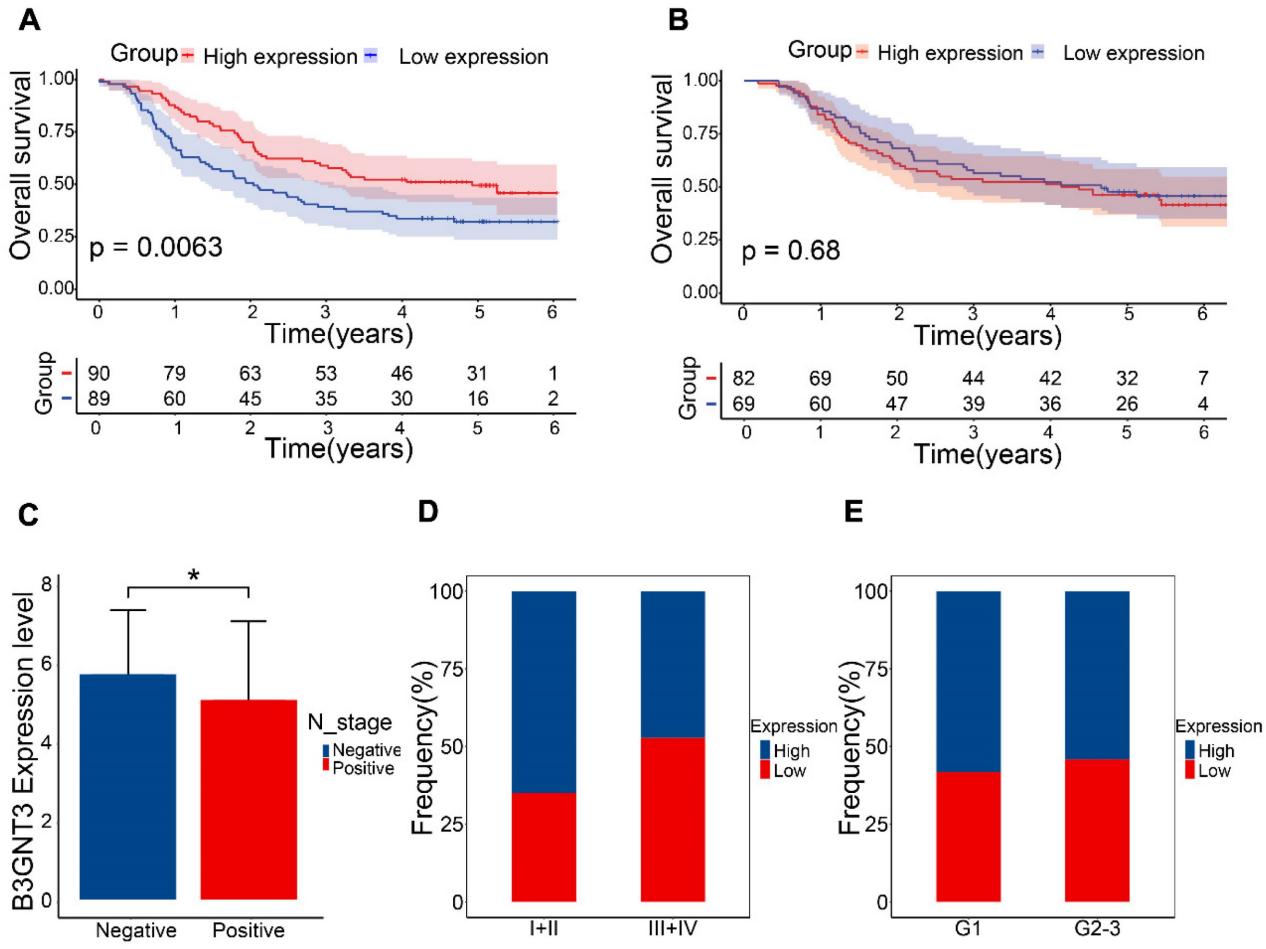
Overall survival analysis of B3GNT3 in ESCC tissues. (A) Kaplan-Meier survival analysis of B3GNT3 in 179 ESCC patients. (B) Kaplan-Meier survival analysis of B3GNT3 from the tissue microarray. (C) show the expression of B3GNT3 according to lymph node metastasis. Stacked bar graphs (D and E) show the percentage of occurrences of patients with high and low expression in pTNM staging and tumor grading. Blue: high expression and red: low expression.

The GEO dataset indicated that the B3GNT3 low expression group exhibited a poorer prognosis (**Figure 7A**), whereas the Kaplan-Meier curves generated from tissue microarrays indicated no statistically significant difference in survival between patients in the high and low expression groups (**Figure 7B**). B3GNT3 expression was lower in patients with positive lymph node metastasis, implying a potential involvement of B3GNT3 in lymph node metastasis (**Figure 7C**). Consistently, a higher proportion of patients with low B3GNT3 expression were found in patients with higher pTNM stage and higher tumor grade (**Figure 7D** and** E**). This suggests that low B3GNT3 expression is associated with multiple risk factors.

### 3.7. Immune cell infiltration and pathway enrichment analysis of B3GNT3

**Figure 8:**
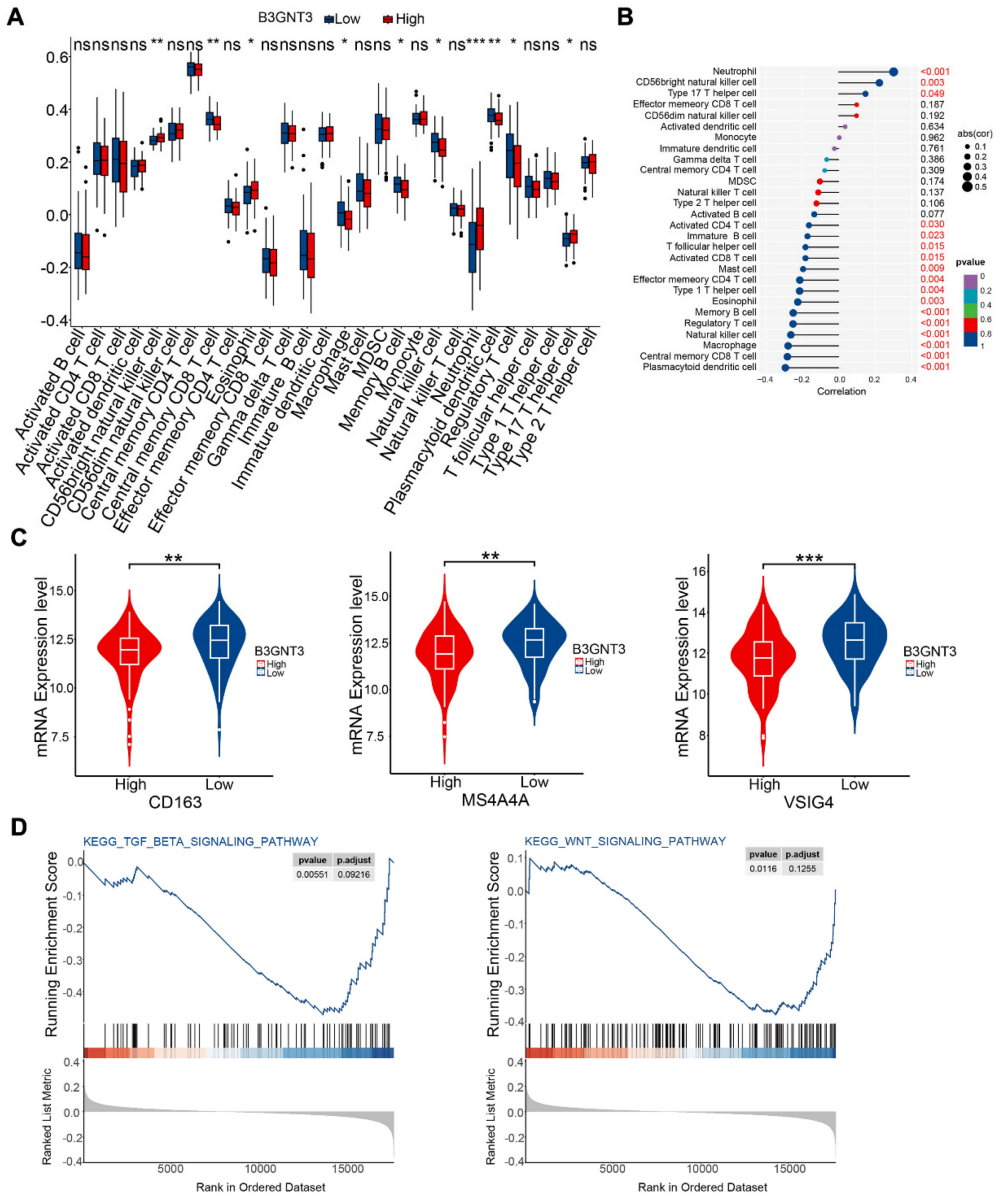
Immune cell infiltration and Gene Set Enrichment Analysis. (A) Immune cell infiltration in the B3GNT3 high and low expression groups. (B) Spearman's correlation between B3GNT3 expression and 28 different immune cells. (C) M2 macrophage cell marker expression in the high and low B3GNT3 expression groups. (D) Enrichment plot of the TGF beta signaling and WNT signaling pathways. The p values are indicated using asterisks (ns, no significantly, * p < 0.05, ** p < 0.01, *** p < 0.001, **** p< 0.0001).

We quantified immune cell infiltration in 179 ESCC patients using ssGSEA to compare the groups with high and low expression of B3GNT3. The results showed a notable enrichment of regulatory T cells, macrophages, and natural killer cells in the low B3GNT3 expression group (**Figure 8A**). Furthermore, Spearman's rank correlation analysis revealed a negative association between B3GNT3 expression and regulatory T cells, macrophages, and natural killer cells (**Figure 8B**). Specifically, the levels of M2 macrophage-associated cellular markers, CD163, MS4A4A, and VSIG4, were significantly elevated in the group with low expression of B3GNT3 (**Figure 8C**). Additionally, GSEA analysis was performed to explore the potential pathways associated with B3GNT3 in immune function, and the results showed a significant enrichment of the TGF-beta signaling pathway and WNT signaling pathway (**Figure 8D**). These findings highlight the crucial role of B3GNT3 in the immune microenvironment.

## 4. Discussion

ESCC is the predominant type of esophageal carcinoma worldwide, and it is associated with high aggressiveness and poor prognosis. Despite the development of individualized precision therapy for ESCA, the 5-year survival rate of ESCC remains below 20% 12, 13. In general, physicians in clinical practice provide guidance regarding follow-up, probability of survival, time to recurrence, and therapeutic schedules for patients based on pTNM stage. However, although pTNM staging systems are valuable in clinical practice, they have limitations in predicting patient prognosis.

Most previous studies have constructed prognostic models based on gene expression. Models combining patients' clinical characteristics and gene expression can provide more information and a more comprehensive understanding of the patient's condition, which can help clinicians develop personalized treatment plans. A stable prognostic model including three genes and two clinical variables was constructed. The model showed better accuracy and predictability than pTNM staging system. Risk stratification analysis further demonstrated the model's clinical applicability, providing valuable insights for clinical decision-making.

B3GNT3, MACC1, and NELL2 have emerged as potential biomarkers for predicting the prognosis of ESCC patients. A study on neuroblastoma indicated a correlation between low expression of B3GNT3 (β-1,3-N-acetylglucosaminyl transferase) and poor prognosis, which aligns with the present results of B3GNT3 RT-PCR and IHC 14. In a study on pancreatic cancer, B3GNT3 was highly expressed in cell lines, but knocking down B3GNT3 expression increased the invasiveness and metastatic capabilities of the cells 15. Previous research showed that B3GNT3 is highly expressed in ESCC cell lines, however, in our cohort, B3GNT3 was downregulated in ESCC tissues compared with normal tissues 16. This contradiction may be attributed to more complex regulatory mechanisms and the influence of the tumor microenvironment on B3GNT3 *in vivo*. Additionally, in the pancreatic cancer study, knocking down B3GNT3 expression resulted in decreased expression of E-cadherin and increased expression of β-catenin, which are key proteins involved in epithelial-mesenchymal transition, promoting tumor cell invasion, metastasis, and resistance to chemotherapy and immunotherapy 15, 17. Similarly, in our cohort, low expression of B3GNT3 was associated with several risk factors, including advanced pTNM stage and a higher probability of lymph node metastasis, which contribute to poor prognosis. The tumor immune microenvironment is one of the main factors associated with malignant progression, and the accumulation of immune infiltrating cells in the tumor microenvironment is associated with cancer progression 18. The potential function of B3GNT3 in the immune microenvironment has been investigated in many cancers 19, 20. The role of B3GNT3 in the immune microenvironment in ESCC remains unknown. In this study, bioinformatics analysis revealed that low expression of B3GNT3 is associated with an increased presence of regulatory T cells, NK cells, and macrophages in ESCC. Specifically, the low B3GNT3 group exhibited more pronounced enrichment of M2 macrophages. Additionally, B3GNT3 expression was correlated with key pathways related to the establishing of an immune-suppressive microenvironment, including TGF-β and WNT signaling 21-23. β-Catenin is a key downstream target of the canonical WNT pathway, and dysregulation of this pathway may disrupt cancer immune surveillance, ultimately promoting immune evasion 24. Enrichment of M2 macrophages enhances the process of epithelial-mesenchymal transition and activates the WNT pathway in various cancer types 25, 26. Therefore, in the context of ESCC, low expression of B3GNT3 may promote the accumulation of M2 macrophages, thereby influencing the epithelial-mesenchymal transition process and WNT pathway, ultimately facilitating tumor cell infiltration and metastasis. This may help explain the correlation between B3GNT3 expression and prognosis in ESCC patients. Further *in vivo* experimental validation is necessary.

Metastasis-associated colon cancer-1 (MACC1) has been identified in various solid tumors and is closely associated with tumor progression, invasion, and metastasis 27. MACC1 serves as an important biomarker and therapeutic target in ESCC, and it promotes invasion and lymph node metastasis primarily through the PTEN/PI3K/Akt and AMPK-ULK1 signaling pathways 28, 29. Zhu *et al.* demonstrated the overexpression of MACC1 in ESCA and its correlation with lymph node metastasis and pTNM staging 29. However, in this study, MACC1 expression was lower in ESCC tissues than in normal tissues in the tissue microarray cohort. This discrepancy could be attributed to differences in grouping methods, as well as variations in the histopathology types used for IHC. Zhu *et al.* used an IRS threshold of 2 to categorize patients into high- and low-expression groups, whereas we used the median IRS for grouping. To validate the significance of MACC1, further refinement of the grouping method and an increase in sample size are necessary.

Nerve epidermal growth factor-like 2 (NELL2) plays a critical role in the development, survival, and activity of neurons in animals 30. NELL2 is significantly downregulated in renal cell carcinoma tissues, which is consistent with the present IHC results 31. Zhang *et al.* constructed an extracellular matrix-based signature using ESCC datasets, including NELL2, IHC analysis demonstrated high expression of NELL2 in ESCC tissues 32. However, in the present cohort, NELL2 exhibited significantly low expression, leading to conflicting results. We speculate that this discrepancy may be due to tumor heterogeneity.

This study has certain limitations and shortcomings. In our cohort, there was no significant difference in the expression of B3GNT3 and prognosis. This could potentially be attributed to post-translational modifications affecting protein activity and expression, although the underlying mechanisms require further investigation. Additionally, there was inconsistency in the expression of MACC1 and NELL2, which may be due to the high heterogeneity of tumor tissues from different sources. Expanding the sample size and including a greater number representative samples would enhance the reliability and generalizability of the study.

## 5. Conclusions

This study performed a comprehensive bioinformatics analysis of the publicly available databases GSE53624 and GSE53622. The results of this analysis were used to construct a stable prognostic model by integrating two clinical features and three genes. Compared with pTNM stage alone, this model demonstrated superior performance as a prognostic predictor in patients with ESCC. B3GNT3, a critical gene included in the model, is associated with the tumor immune microenvironment and the prognosis of ESCC. This study introduced a novel signature for prognostic prediction in ESCC, which may help the development of personalized treatment strategies. The study also provides an important foundation for further research on B3GNT3 and the immune microenvironment in ESCC.

## Supplementary Material

Supplementary tables.

## Figures and Tables

**Table 1:** Primer sequences for RT-PCR

Primer	Forward (5'-3')	Reverse (5'-3')
B3GNT3	GGACTTCCACGACTCCTTCTTC	GCACCTTGTCTCCTGCCACT
MACC1	CTTAGACCAGGCAATCATTACGG	GCCCAGCAGTCTGTTTCACC
NELL2	GAAGGGAACCACCTACCGAG	CACATACGCAAGAGCCGACT
β-ACTIN	CATCGAGCACGGCATCGTCA	TAGCACAGCCTGGATAGCAAC

**Table 2:** The 24 prognostic genes identified in the two subsets.

Gene	GSE53624		GSE53622	
	p value	HR	p value	HR
B3GNT3	0.030	0.601	0.034	0.476
BDH1	0.046	0.624	0.007	0.389
C6orf226	0.041	0.618	0.047	0.492
CHD9	0.048	1.589	0.007	2.627
DIPK1B	0.012	1.807	0.011	2.460
DZIP1	0.006	1.895	0.014	2.379
HECTD2	0.017	1.751	0.014	2.385
HSD17B1	0.012	0.553	0.023	0.448
MACC1	0.008	0.534	0.037	0.482
MANF	0.040	0.617	0.040	0.487
MON1A	0.048	0.628	0.026	0.455
MPDZ	0.004	1.987	0.037	2.078
NELL2	0.031	0.602	0.001	0.322
NKAIN1	0.007	0.530	0.015	0.424
NLRP6	0.049	0.628	0.029	0.460
NOTCH2NLC	0.026	1.690	0.007	2.627
PIGT	0.015	1.773	0.006	2.694
PIN4	0.037	1.630	0.005	2.753
PLA2G4A	0.011	0.549	0.027	0.456
PYGB	0.042	1.613	0.036	2.083
SRSF6	0.004	1.955	0.004	2.791
STX2	0.014	1.777	0.014	2.418
USH1G	0.033	0.605	0.045	0.496
WEE2	0.024	1.699	0.008	2.600
