# Supplementary material for: Bioinformatics Analysis Reveals a Novel Prognostic Model for Esophageal Squamous Cell Carcinoma
Source: Int J Med Sci. 2024 May 5;21(7):1213–26. doi: 10.7150/ijms.93423 (PMC11134584; doi:10.7150/ijms.93423)
Supplement: Supplementary file 1 — Supplementary tables. [file ijmsv21p1213s1.pdf]

Table S1: Demographics and characteristics of 179 patients from GEO.

**Characteristics** [a]	**Total (N=179)**
**Age**	
<60	91 (50.8%)
≥60	88 (49.2%)
**Gender**	
Male	146 (81.6%)
Female	33 (18.4%)
**Alcohol use**	
Yes	106 (59.2%)
No	73 (40.8%)
**Tumor grade**	
G1	49 (27.4%)
G2	98 (54.7%)
G3	32 (17.9%)
**T stage**	
T1	12 (6.7%)
T2	27 (15.1%)
T3	110 (61.5%)
T4	30 (16.8%)
**N stage**	
N0	83 (46.4%)
N1	62 (34.6%)
N2	22 (12.3%)
N3	12 (6.7%)
**Stage**	
I	7 (3.9%)
II	71 (39.7%)
III	85 (47.5%)
IV	16 (8.9%)

[a] N stage, T stage and Stage were determined according to American Joint Committee on Cancer 8th edition.

Table S2: Detailed clinical characteristics of 10 Patients for RT-PCR.

ID	Age	Sex	Pathological type	Tumor Grade	T stage	N stage	Stage
P1	67	male	ESCC	G2	T3	N0	II
P2	55	male	ESCC	G2	T3	N1	III
P3	62	male	ESCC	G3	T3	N2	III
P4	65	female	ESCC	G1	T1	N0	I
P5	74	male	ESCC	G3	T3	N0	II
P6	54	male	ESCC	G1	T3	N1	III
P7	66	male	ESCC	G3	T2	N0	I
P8	62	male	ESCC	G3	T2	N0	I
P9	60	male	ESCC	G1	T3	N1	III
P10	63	male	ESCC	G2	T3	N0	II

[a] N stage, T stage and Stage were determined according to American Joint Committee on Cancer 8th edition

Table S3: Demographic characteristics of the 154 patients used for IHC.

Characteristics [a]	**Total (N=154)**
**Age**	
<60	62 (40.3%)
≥60	92 (59.7%)
**Gender**	
female	25 (16.2%)
male	129 (83.8%)
**Alcohol use**	
Yes	99 (64.3%)
No	55 (35.7%)
**Tumor grade**	
G1	12 (7.8%)
G2	78 (50.6%)
G3	64 (41.6%)
**T stage**	
T1	8 (5.2%)
T2	25 (16.2%)
T3	121 (78.6%)
**N stage**	
N0	61 (39.6%)
N1	59 (38.3%)
N2	26 (16.9%)
N3	8 (5.2%)
**Stage**	
I	14 (9.1%)
II	49 (31.8%)
III	83 (53.9%)
IV	8 (5.2%)

[a] N stage, T stage and Stage were determined according to American Joint Committee on Cancer 8th edition.
